# Supplementary material for: Extracellular miRNAs as Predictive Biomarkers for Glypican-3-Derived Peptide Vaccine Therapy Response in Ovarian Clear Cell Carcinoma
Source: Cancers (Basel). 2021 Feb 1;13(3):550. doi: 10.3390/cancers13030550 (PMC7867082; doi:10.3390/cancers13030550)
Supplement: Supplementary file 1 [file cancers-13-00550-s001.pdf]

Table S1. Responsive > unresponsive in remission group (32 miRNAs of absolute fold change > 2)

	Responsive						Unresponsive							Average (R1-5, R7)	Average (P8-14)	Fold change {Average(R1-5,R7)+1/Average(R8-14)+1}
	R1	R2	R3	R4	R5	R7	R8	R9	R10	R11	R12	R13	R14			
miR-4497	104	19	0	0	0	26	0	0	0	0	0	0	0	25	0	25.80
miR-365a-3p	0	9	0	0	23	129	0	6	0	0	0	8	0	27	2	9.26
miR-206	0	206	160	67	196	8559	0	199	60	556	298	264	113	1531	213	7.17
miR-193b-5p	139	47	534	67	60	52	14	35	23	19	23	33	30	150	25	5.76
miR-1228-5p	35	37	445	80	53	233	32	41	23	19	23	33	15	147	26	5.39
miR-433-3p	104	9	53	13	0	0	9	0	0	19	0	8	0	30	5	5.07
miR-365a-5p	139	19	36	0	23	0	0	0	0	37	0	16	0	36	8	4.28
miR-139-3p	660	280	534	308	286	543	46	164	60	74	140	156	105	435	107	4.06
miR-485-5p	139	47	71	27	45	78	0	41	8	37	18	25	8	68	19	3.38

miR-1-3p	35	224	36	13	75	6361	27	468	45	723	269	371	436	1124	334	3.36
miR-193a-5p	1217	1477	1655	777	467	1319	370	567	280	408	315	321	286	1152	364	3.16
miR-483-5p	487	93	498	147	136	207	110	70	38	93	47	82	135	261	82	3.16
miR-122-5p	161314	48863	357597	107942	70872	83911	5282	47704	71425	56987	64655	44617	44182	138416	47836	2.89
miR-874-3p	0	19	0	0	8	129	18	23	0	0	0	16	0	26	8	2.89
miR-320c	660	439	1264	255	249	362	242	199	159	278	146	206	75	538	186	2.88
miR-122b-3p	68891	22674	117995	48332	21073	31134	3009	19174	23163	25424	21762	19072	16802	51683	18344	2.82
miR-184	209	159	89	121	53	5689	55	269	15	538	257	288	1210	1053	376	2.80
miR-320d	174	65	249	67	30	52	32	35	8	93	6	74	23	106	39	2.71
miR-23a-5p	0	56	107	54	0	26	23	23	0	19	0	8	30	40	15	2.63
miR-150-5p	313	290	160	737	429	1060	137	117	144	74	228	206	459	498	195	2.55
miR-99b-3p	70	65	107	94	8	78	27	64	30	0	35	16	23	70	28	2.45
miR-	3163	2290	3559	1326	949	1241	1043	1151	582	1131	666	988	556	2088	874	2.39

320b																
miR-190b-5p	0	19	0	0	23	181	14	6	8	37	18	16	8	37	15	2.36
miR-505-3p	0	19	18	13	45	129	14	6	15	37	12	16	8	37	15	2.35
miR-6852-5p	174	47	18	40	23	129	18	64	15	0	41	66	8	72	30	2.33
miR-127-3p	626	93	231	429	105	1603	101	228	318	204	309	288	105	515	222	2.31
miR-92b-5p	209	37	142	67	128	26	59	0	106	19	82	25	15	102	44	2.30
miR-375-3p	5005	1748	1388	3282	2818	3051	375	742	1444	1317	2190	1581	1398	2882	1292	2.23
miR-1273h-3p	35	84	107	54	45	103	18	23	8	56	12	74	45	71	34	2.09
miR-181a-3p	0	47	107	0	0	0	18	18	0	0	0	16	30	26	12	2.08
miR-4433a-3p	243	112	89	40	30	362	73	64	0	148	29	156	30	146	72	2.03
miR-146b-3p	104	9	53	40	30	78	5	41	0	56	6	41	30	52	25	2.02

Table S2. Unresponsive > responsive in remission group (45 miRNAs of absolute fold change < 0.5)

	Responsive						Unresponsive							Average (R1-5, R7)	Average (P8-14)	Fold change {Average(R1-5,R7)+1/Average(R8-14)+1}
	R1	R2	R3	R4	R5	R7	R8	R9	R10	R11	R12	R13	R14			
miR-106b-5p	70	65	53	27	121	52	183	82	121	56	93	140	256	65	133	0.49
miR-140-3p	1495	2365	819	1755	1740	1034	4706	3705	2102	2429	2178	2841	4150	1535	3159	0.49
miR-421	0	75	0	27	60	26	27	105	45	148	41	99	8	31	68	0.47
miR-200c-3p	0	28	18	54	38	26	18	47	38	93	29	124	68	27	59	0.47
miR-148b-3p	1425	1561	730	1474	1439	1862	2177	3898	1641	4543	1693	3516	3849	1415	3045	0.46
miR-576-3p	104	84	18	27	23	0	23	99	23	204	53	140	113	43	93	0.46
miR-200a-3p	70	19	18	147	75	78	23	99	45	519	64	264	23	68	148	0.46
miR-107	35	196	89	107	196	103	544	146	242	223	210	165	391	121	274	0.44
miR-143-3p	1217	4785	1264	6149	2094	6956	3576	9730	2488	16486	4052	11793	11284	3744	8487	0.44

miR-101-3p	2120	2393	356	2237	2471	802	3453	4015	3320	3449	3106	3590	7149	1730	4012	0.43
miR-425-3p	0	37	107	80	15	26	169	140	38	148	41	107	90	44	105	0.43
miR-20a-5p	209	430	18	268	678	362	572	766	824	371	794	651	1526	327	786	0.42
miR-30e-5p	1008	2075	1014	2291	2705	2301	3018	5932	4280	4673	4187	4875	5473	1899	4634	0.41
miR-186-5p	452	1794	1086	1500	1650	724	1907	3495	2306	2726	2511	3475	4270	1201	2956	0.41
miR-15b-3p	35	28	0	40	113	52	220	99	113	130	88	58	83	45	113	0.40
miR-126-5p	174	122	53	94	181	155	224	187	151	686	169	404	466	130	327	0.40
miR-19b-3p	70	93	53	13	151	52	325	275	113	167	117	173	158	72	190	0.38
miR-16-5p	348	1131	107	1085	1846	853	5634	1823	1528	1131	1588	1120	3924	895	2393	0.37
miR-1290	104	47	214	13	38	103	114	76	83	760	93	387	135	87	236	0.37
miR-16-2-3p	313	879	356	804	1582	672	1518	2174	3244	1409	2972	1861	1376	768	2079	0.37
miR-3613-5p	0	37	36	40	30	78	165	70	76	111	88	82	128	37	103	0.36

miR-130b-5p	35	19	36	0	38	52	55	129	45	167	35	140	15	30	84	0.36
miR-450a-5p	0	56	0	40	30	78	9	82	68	241	76	140	53	34	96	0.36
miR-142-5p	70	252	107	295	362	233	521	549	492	909	473	527	782	220	608	0.36
miR-27a-3p	1112	2626	570	2425	1552	4215	1610	5143	3002	16449	2914	9182	3233	2083	5933	0.35
miR-19a-3p	0	47	36	40	121	26	101	181	136	167	111	107	120	45	132	0.34
miR-548o-3p	0	28	0	13	8	78	14	70	53	148	41	99	38	21	66	0.33
miR-340-5p	70	355	53	241	241	310	174	660	287	1484	286	947	707	212	649	0.33
miR-450b-5p	0	37	53	13	8	0	0	29	30	130	47	82	98	19	59	0.32
miR-142-3p	70	65	18	27	113	26	37	210	151	371	134	214	83	53	171	0.31
miR-582-3p	0	56	18	94	8	103	41	269	45	389	47	198	83	46	153	0.31
miR-3168	104	0	231	0	8	0	96	263	454	74	350	99	45	57	197	0.29
miR-203b-5p	104	150	71	40	68	78	50	111	174	56	485	898	316	85	298	0.29

miR-96-5p	0	0	0	40	38	0	32	35	106	19	82	16	53	13	49	0.28
miR-203a-3p	104	131	53	40	90	26	46	105	212	37	502	807	278	74	284	0.26
miR-144-3p	35	122	53	134	392	0	402	193	1233	148	969	148	346	123	491	0.25
miR-32-5p	0	47	0	0	38	26	50	58	68	185	64	91	45	18	80	0.24
miR-374a-5p	35	47	0	0	60	0	114	76	106	185	82	91	158	24	116	0.21
miR-199b-5p	0	56	0	27	15	26	18	146	45	278	47	173	30	21	105	0.20
miR-151a-5p	0	28	178	13	83	0	540	64	197	315	158	214	323	50	259	0.20
miR-15a-5p	0	0	0	40	15	52	302	29	45	56	47	41	165	18	98	0.19
let-7a-3p	0	0	0	54	68	103	96	82	242	464	216	288	233	37	232	0.17
miR-34c-5p	0	0	0	0	0	26	0	0	121	0	93	0	23	4	34	0.15
miR-9-3p	0	0	0	0	0	0	0	0	121	19	93	8	15	0	37	0.03
miR-9-5p	0	28	0	13	15	0	5	12	2722	0	2102	8	391	9	749	0.01

Table S3. Responsive > unresponsive in progression group (48 miRNAs of absolute fold change > 2)

	Responsive		Unresponsive					Average (P1, P2)	Average (P5-8, P10)	Fold change {Average(P1, P2)+1/Average(P5-8, P10)+1}
	P1	P2	P5	P6	P7	P8	P10			
miR-499a-5p	0	229	0	0	0	0	0	115	0	115.54
miR-6855-5p	0	115	0	0	0	0	0	57	0	58.27
miR-409-5p	113	0	0	0	0	0	0	56	0	57.38
miR-4718	113	0	0	0	0	0	0	56	0	57.38
miR-1306-5p	113	115	0	18	0	0	0	114	4	24.61
miR-3605-5p	169	0	0	37	0	0	0	85	7	10.29
miR-339-5p	56	458	0	55	0	150	0	257	41	6.14
miR-542-3p	56	172	83	18	0	0	0	114	20	5.41
miR-660-5p	338	115	0	55	0	150	0	226	41	5.40
miR-133a-3p	0	344	0	18	0	150	0	172	34	4.97
miR-125b-5p	1804	573	498	37	214	376	182	1188	261	4.53
miR-204-3p	113	0	0	0	0	0	61	56	12	4.38
miR-151a-5p	56	458	166	18	0	0	121	257	61	4.16

miR-3613-5p	282	229	0	18	0	226	61	255	61	4.14
miR-28-5p	0	115	0	0	71	0	0	57	14	3.82
miR-215-5p	620	115	0	37	143	301	0	367	96	3.80
miR-2355-3p	113	0	0	0	71	0	0	56	14	3.76
miR-9-5p	113	57	0	37	0	75	0	85	22	3.68
miR-320c	5751	172	415	146	1355	903	1331	2961	830	3.56
miR-29a-3p	3214	573	415	274	428	978	666	1893	552	3.42
miR-375-3p	2706	2119	581	329	1569	677	363	2413	704	3.42
miR-194-5p	1128	401	166	238	143	451	121	764	224	3.40
miR-1228-5p	226	286	0	91	143	150	0	256	77	3.30
miR-10b-3p	113	172	0	0	0	226	0	142	45	3.10
miR-543	0	115	0	18	0	75	0	57	19	2.96
miR-335-5p	113	172	0	18	0	226	0	142	49	2.88
miR-22-3p	15786	4753	3736	1646	2996	6321	3207	10270	3581	2.87
miR-320b	10374	1203	1744	787	2853	1806	3146	5788	2067	2.80
miR-130a-3p	113	57	0	0	0	150	0	85	30	2.77
miR-5010-5p	56	115	83	18	0	0	61	85	32	2.59
miR-126-5p	451	687	498	18	71	602	0	569	238	2.39

miR-4508	395	344	166	274	0	150	182	369	154	2.38
miR-125a-5p	395	1145	830	91	285	301	121	770	326	2.36
miR-193a-5p	1973	2291	1079	750	499	1204	1089	2132	924	2.31
miR-942-5p	0	229	0	55	0	75	121	115	50	2.26
miR-206	56	802	664	0	285	0	0	429	190	2.25
miR-382-5p	169	229	0	110	214	75	61	199	92	2.15
miR-320a-3p	69516	17811	24078	10995	14337	20767	32490	43663	20533	2.13
miR-3074-5p	3552	1432	2242	622	428	2182	484	2492	1192	2.09
miR-23a-5p	0	115	0	0	0	75	61	57	27	2.07
miR-625-3p	169	401	83	201	0	226	182	285	138	2.05
miR-107	282	344	166	91	143	301	61	313	152	2.05
miR-500a-3p	395	115	166	55	143	75	182	255	124	2.04
miR-23b-3p	507	344	332	293	71	226	121	426	209	2.04
miR-140-5p	169	458	249	146	0	376	0	314	154	2.03
miR-130b-5p	0	115	0	18	0	0	121	57	28	2.02
let-7a-3p	169	115	166	37	71	75	0	142	70	2.02
miR-150-5p	113	1145	581	329	0	527	121	629	312	2.02

Table S4. Unresponsive > responsive in progression group (61 miRNAs of absolute fold change < 0.5)

	Responsive		Unresponsive					Average (P1, P2)	Average (P5-8, P10)	Fold change {Average(P1, P2)+1/Average(P5-8, P10)+1}
	P1	P2	P5	P6	P7	P8	P10			
miR-203b-5p	56	172	747	18	143	226	61	114	239	0.48
miR-576-5p	0	57	83	0	71	150	0	29	61	0.48
miR-10a-3p	56	0	0	0	0	301	0	28	60	0.48
miR-148a-3p	27851	36997	47908	95220	109130	41159	50036	32424	68690	0.47
miR-122-5p	49727	36080	15692	79890	116120	66215	184414	42903	92466	0.46
miR-335-3p	0	57	83	18	143	75	0	29	64	0.46
miR-30a-3p	226	57	1245	73	71	150	61	141	320	0.44
miR-200a-3p	507	57	1744	55	642	752	61	282	651	0.43
miR-145-5p	0	57	166	37	0	75	61	29	68	0.43
miR-15a-5p	56	0	83	55	71	150	0	28	72	0.40
miR-17-5p	113	115	498	146	214	451	121	114	286	0.40
miR-127-3p	0	515	0	1079	1498	376	303	258	651	0.40
miR-196b-5p	0	57	332	37	0	0	0	29	74	0.40

miR-3158-3p	56	172	83	585	285	75	424	114	291	0.39
miR-345-5p	169	0	415	165	0	226	303	85	222	0.38
miR-125a-3p	0	57	0	55	214	0	121	29	78	0.38
miR-671-3p	0	57	166	55	0	75	121	29	83	0.35
miR-3529-3p	113	344	332	677	1427	527	303	228	653	0.35
miR-20b-5p	56	0	83	37	71	226	0	28	83	0.35
miR-7-5p	226	229	581	677	1427	602	121	227	682	0.33
miR-365a-5p	0	57	0	18	214	150	61	29	89	0.33
miR-1246	1240	2749	7971	1756	17190	1053	5748	1995	6744	0.30
miR-7704	56	0	249	37	214	0	0	28	100	0.29
miR-200c-3p	677	115	5563	128	428	752	0	396	1374	0.29
miR-339-3p	0	115	249	220	0	150	424	57	209	0.28
miR-210-3p	0	57	83	37	214	75	121	29	106	0.28
miR-200b-3p	282	115	3902	91	214	301	0	198	902	0.22
miR-1843	0	57	249	128	71	226	0	29	135	0.22
miR-664a-5p	56	0	249	165	0	75	242	28	146	0.20
miR-429	56	0	664	0	71	75	0	28	162	0.18
miR-4488	56	0	332	73	214	301	0	28	184	0.16

miR-203a-3p	56	115	2823	37	143	301	61	85	673	0.13
miR-1290	282	115	2740	421	5635	226	1634	198	2131	0.09
miR-184	56	0	1827	18	357	75	363	28	528	0.06
miR-6511b-5p	0	0	0	0	0	0	121	0	24	0.04
miR-6734-3p	0	0	0	0	0	0	121	0	24	0.04
miR-3690	0	0	0	0	143	0	0	0	29	0.03
miR-708-3p	0	0	0	0	143	0	0	0	29	0.03
miR-17-3p	0	0	0	0	0	150	0	0	30	0.03
miR-505-3p	0	0	0	0	0	150	0	0	30	0.03
miR-455-5p	0	0	0	18	143	0	0	0	32	0.03
miR-296-5p	0	0	166	0	0	0	0	0	33	0.03
miR-5588-5p	0	0	166	0	0	0	0	0	33	0.03
miR-1304-3p	0	0	0	0	0	75	121	0	39	0.02
miR-363-5p	0	0	83	0	0	0	121	0	41	0.02
miR-92b-5p	0	0	0	37	0	0	182	0	44	0.02
miR-340-3p	0	0	83	0	0	150	0	0	47	0.02
miR-1269a	0	0	249	0	0	0	0	0	50	0.02
miR-485-5p	0	0	0	37	143	75	0	0	51	0.02

miR-454-5p	0	0	166	18	71	0	0	0	51	0.02
miR-574-5p	0	0	83	55	0	0	121	0	52	0.02
miR-1273h-5p	0	0	0	18	0	0	242	0	52	0.02
miR-200a-5p	0	0	249	0	71	0	0	0	64	0.02
miR-200b-5p	0	0	249	18	0	0	61	0	66	0.02
miR-1307-5p	0	0	0	37	71	226	0	0	67	0.01
miR-370-3p	0	0	0	220	71	0	61	0	70	0.01
miR-381-3p	0	0	0	256	143	0	0	0	80	0.01
miR-582-3p	0	0	166	183	0	0	121	0	94	0.01
miR-148a-5p	0	0	83	91	143	0	182	0	100	0.01
miR-3168	0	0	0	256	0	0	666	0	184	0.01
miR-4433b-3p	0	0	166	165	214	301	242	0	218	0.00
